# Glucose Metabolism and Oxygen Availability Govern Reactivation of the Latent Human Retrovirus HTLV-1

**DOI:** 10.1016/j.chembiol.2017.08.016

**Published:** 2017-11-16

**Authors:** Anurag Kulkarni, Manuel Mateus, Cyrille C. Thinnes, James S. McCullagh, Christopher J. Schofield, Graham P. Taylor, Charles R.M. Bangham

**Affiliations:** 1 Section of Virology, Department of Medicine, Imperial College, London W2 1PG, UK; 2 Chemistry Research Laboratory, Department of Chemistry, University of Oxford, Mansfield Road, Oxford, UK

**Keywords:** HTLV-1, latency, virus, metabolism, hypoxia, glucose, 2-oxoglutarate, HIF hydroxylase, epigenetic regulation

## Abstract

The human retrovirus HTLV-1 causes a hematological malignancy or neuroinflammatory disease in ∼10% of infected individuals. HTLV-1 primarily infects CD4^+^ T lymphocytes and persists as a provirus integrated in their genome. HTLV-1 appears transcriptionally latent in freshly isolated cells; however, the chronically active anti-HTLV-1 cytotoxic T cell response observed in infected individuals indicates frequent proviral expression *in vivo*. The kinetics and regulation of HTLV-1 proviral expression *in vivo* are poorly understood. By using hypoxia, small-molecule hypoxia mimics, and inhibitors of specific metabolic pathways, we show that physiologically relevant levels of hypoxia, as routinely encountered by circulating T cells in the lymphoid organs and bone marrow, significantly enhance HTLV-1 reactivation from latency. Furthermore, culturing naturally infected CD4^+^ T cells in glucose-free medium or chemical inhibition of glycolysis or the mitochondrial electron transport chain strongly suppresses HTLV-1 plus-strand transcription. We conclude that glucose metabolism and oxygen tension regulate HTLV-1 proviral latency and reactivation *in vivo*.

## Highlights

• Physiological (1%) hypoxia enhances HTLV-1 plus-strand transcription
• HTLV-1 transcription is hypoxia regulated but HIF independent
• Inhibition of glycolysis or the mitochondrial ETC suppresses HTLV-1 transcription
• Extracellular glucose concentration regulates HTLV-1 reactivation from latency

## Graphical Abstract

**Figure:**
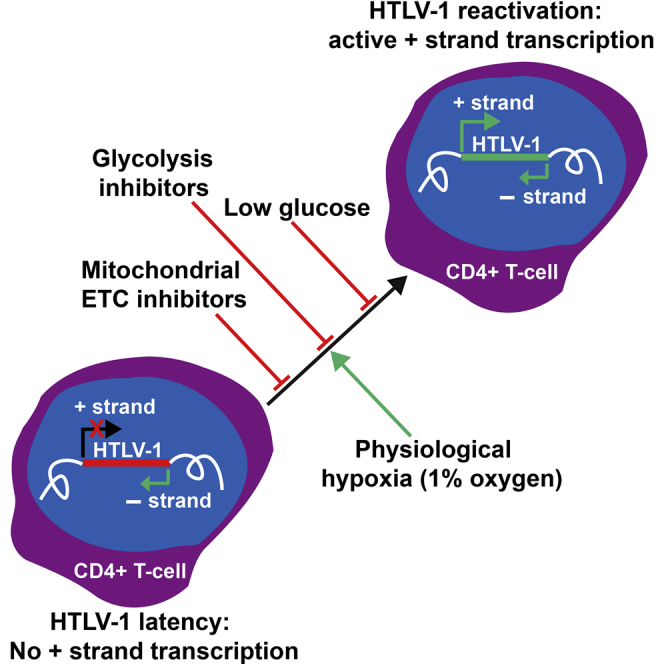

## Teaser

The human leukemia virus HTLV-1 remains dormant most of the time in the infected person, but is intermittently reactivated by unknown mechanisms. Kulkarni et al. show that fluctuations in glucose metabolism and oxygen availability are two major factors that govern the reactivation of HTLV-1 from dormancy.

## Introduction

HTLV-1 is a human retrovirus that primarily infects CD4^+^ T lymphocytes. It is a single-stranded positive-sense RNA virus that reverse transcribes its RNA genome and integrates the resulting double-stranded DNA copy of its genome into the host cellular chromatin (as a provirus), thereby establishing a persistent infection in the cells. It is estimated that 5–10 million people worldwide are HTLV-1 carriers. HTLV-1 infection is asymptomatic in most cases. In a subset of infected individuals (∼5%–10%), however, HTLV-1 infection progresses to either a CD4^+^ T cell malignancy known as adult T cell leukemia/lymphoma or to HTLV-1-associated myelopathy, a progressive inflammatory disease of the spinal cord.

HTLV-1 infection is considered to be largely latent in infected individuals, because the viral structural RNA and protein products are usually undetectable in freshly isolated infected peripheral blood mononuclear cells (PBMCs) (Hanon et al., 2000, Rende et al., 2011). However, the presence of a sustained chronically activated cytotoxic T cell response against HTLV-1 antigens, in infected individuals, suggests that the immune system frequently encounters newly synthesized viral antigens within the body (Bangham, 2009, Hanon et al., 2000). Hence it is important to understand the mechanisms that regulate the latency, reactivation, and productive infection of HTLV-1 *in vivo*. This understanding might suggest strategies to reactivate the dormant virus and make it accessible to attack by the immune system and antiretroviral drugs.

Previous work on HTLV-1 proviral latency and reactivation has largely focused on identifying the viral factors involved, such as the HTLV-1 integration site (Melamed et al., 2013). Spontaneous reactivation of the provirus is associated with integration in transcriptionally active euchromatin, in opposite transcriptional orientation of the nearest host gene and within 100 bases of certain transcription factor binding sites (Gillet et al., 2011, Melamed et al., 2013). Recently, it has been shown that the HTLV-1 provirus binds the key host chromatin insulator binding transcription factor CTCF (CCCTC binding factor) (Satou et al., 2016). The functional consequences of this binding are not yet clear; we hypothesize that CTCF binding regulates proviral transcription, local chromatin structure, and alternative splicing of mRNA.

Different cell types are exposed to widely differing oxygen tension *in vivo*. While pulmonary alveolar cells experience oxygen concentrations of ∼15%, embryonic, neural, mesenchymal, and hematopoietic stem cells are exposed to profoundly hypoxic environments (∼1% O_2_) (Ivanovic, 2009, Mohyeldin et al., 2010). Viruses have evolved different strategies either to counter the detrimental effects of oxygen variations or to exploit hypoxic cellular metabolism to their own advantage, thereby enabling them to infect different cell types and replicate or persist in the host (Ebbesen and Zachar, 1998, Morinet et al., 2013). Circulating CD4^+^ T cells are of special interest as they encounter frequent changes in oxygen tension and extracellular fluid, and contact several different cell types. We set out to test the hypothesis that these extracellular stresses and changing microenvironment influence transcription of the HTLV-1 provirus. To this end we took two complementary approaches. First, in a “top-down” approach we studied the effects on HTLV-1 transcription of cellular stresses routinely encountered within the body such as physiological hypoxia and nutrient limitation. Second, in a “bottom-up” approach we identified specific epigenetic and transcriptional changes in the provirus in response to specific inhibitors of cellular metabolic and stress-response pathways that are known to play an important role in cellular adaptation to the external environment.

To minimize artifacts due to *in vitro* selection and adaptation, we employed primary PBMCs isolated from HTLV-1-infected individuals to study the effects of various stresses and inhibitors on HTLV-1 reactivation from latency. Plus-strand HTLV-1 gene expression is typically undetectable in fresh PBMCs obtained from HTLV-1-infected individuals, but there is a strong spontaneous increase in the expression of Tax, the viral transactivator of plus-strand gene transcription, within a few hours of isolation (Hanon et al., 2000, Rende et al., 2011). An increase in Tax transcription can also be observed in cultures of whole blood obtained from HTLV-1-infected individuals (Hanon et al., 2000). These observations suggest that changes in the extracellular microenvironment have an important impact on HTLV-1 proviral transcription.

## Results

### Hypoxia Enhances HTLV-1 Plus-Strand Transcription

**Figure 1:**
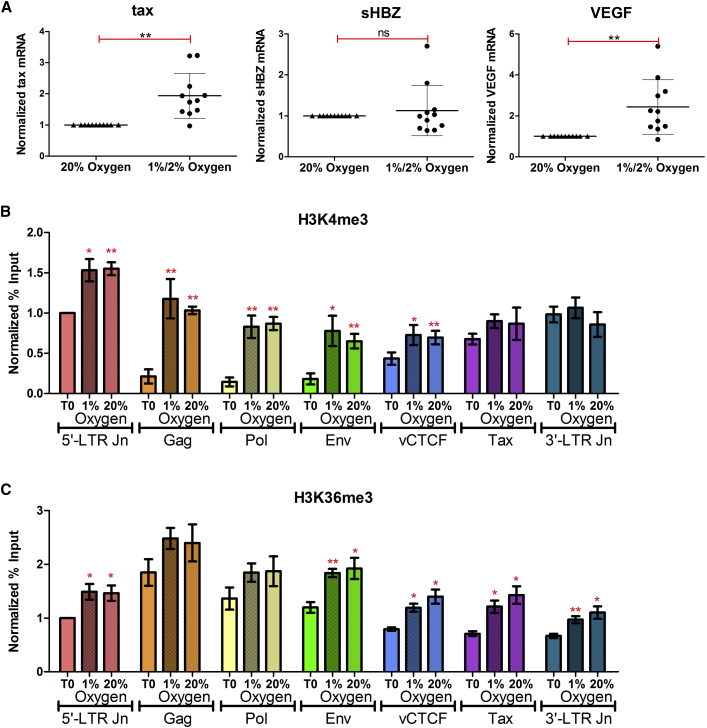
Transcriptional and Epigenetic Impact of Physiological Hypoxia on the HTLV-1 Provirus (A) Hypoxia enhances plus-strand HTLV-1 (*tax*) transcription. PBMCs isolated from HTLV-1-infected individuals (n = 11) were cultured overnight either under atmospheric oxygen conditions (20% oxygen) or under hypoxia (1% or 2% oxygen). RNA was extracted and subjected to qRT-PCR with primers specific for *tax* mRNA (plus strand), *sHBZ* mRNA (minus strand), or *VEGF* mRNA (positive control). Error bars represent the SEM. Statistical significance was calculated using the two-tailed Student's t test (**p < 0.005; ns, not significant). (B and C) Epigenetic changes at the HTLV-1 provirus upon reactivation under physiological hypoxia or normoxia. HTLV-1-infected PBMCs were either fixed immediately (T_0_) or cultured overnight under hypoxia (1% oxygen) or normoxia (20% oxygen) and subsequently fixed and subjected to ChIP-qPCR, using antibodies directed against H3K4me3 (B), H3K36me3 (C), and immunoglobulin G, and primers specific for the 5′-LTR Jn, *Gag*, *Pol*, *Env*, *vCTCF*, *Tax*, and 3′-LTR Jn of the HTLV-1 LTR. Enrichment is expressed as percent input DNA and normalized to T_0_ at the 5′-LTR junction. Error bars represent the SE of four independent ChIP experiments. Statistical significance was calculated using the two-tailed Student's t test (*p < 0.05, **p < 0.005).

The concentration of oxygen in venous blood ranges from 5% to 10%, and in air at sea level is ∼20%. Previous studies of gene expression in HTLV-1-infected patients' PBMCs have been carried out under ambient atmospheric conditions (∼20% oxygen). However, lymphocytes spend most of their time in the highly hypoxic environment of the lymph circulation or solid lymphoid organs (1%–2% oxygen) (Hangai-Hoger et al., 2007, Tsai et al., 2004). To study the impact of physiological hypoxia on the integrated HTLV-1 virus in naturally infected cells, we cultured primary HTLV-1-infected PBMCs overnight either under physiologically relevant hypoxia (1% or 2% oxygen) or normoxia (∼20% oxygen). RNA was then extracted and subjected to qPCR with primers specific for HTLV-1 *tax* (surrogate for HTLV-1 plus-strand transcription), HTLV-1 *sHBZ* (surrogate for minus-strand HTLV-1 transcription), and host cellular *VEGF* (hypoxia-inducible positive control). There was a significant increase in plus-strand transcription in PBMCs cultured under physiological hypoxia when compared with PBMCs cultured under ambient oxygen conditions (Figure 1A). No such change was seen in *sHBZ* transcription levels. As expected, there was also a significant upregulation in transcription of the *VEGF* gene, which is regulated by the hypoxia-induced transcription factor (HIF) (Figure 1A).

### Epigenetic Histone Modifications at the HTLV-1 Provirus upon Reactivation under Normoxia and Physiological Hypoxia

Next, we tested whether the hypoxia-mediated induction of HTLV-1 plus-strand transcription is associated with a specific epigenetic signature in the provirus, by analyzing changes in histone methylation status. HTLV-1-infected frozen PBMCs were thawed and either fixed immediately or cultured overnight under either hypoxia (1% oxygen) or normoxia (20% oxygen), and fixed subsequently at 17 hr. Chromatin from the respective samples was fragmented by sonication and subjected to chromatin immunoprecipitation (ChIP), using antibodies directed against H3K4me3, which is associated with promoters and enhancers; H3K36me3, which is associated with actively transcribed gene bodies; and H3K27me3, a repressive epigenetic mark associated with heterochromatin.

After overnight incubation, H3K4me3 was observed to be significantly enriched at the 5′-LTR (long terminal repeat) junction, *gag*, *pol*, *env*, and *vCTCF* regions of the HTLV-1 provirus in cells cultured in 1% as well as 20% oxygen. By contrast, there was no significant change (within the limits of detection) in H3K4me3 at the 3′-LTR junction (Figure 1B). Similarly, levels of the H3K36me3 mark, which corresponds with proviral activation, were increased across all the regions of the HTLV-1 provirus under physiological hypoxia as well as normoxia (Figure 1C). However, the increase observed in the *gag* and *pol* regions was not statistically significant, perhaps because these regions were highly enriched in the H3K36me3 mark even at time 0 in immediately fixed PBMCs (Figure 1C). Although there was a slight increase in H3K27me3 levels in all the tested regions of the HTLV-1 provirus upon proviral activation under 1% or 20% oxygen, this increase was statistically significant only in the 3′-LTR junction region (Figure S1). However, the apparent change in H3K27me3 status was not associated with any perturbation in minus-strand HTLV-1 transcriptional activity in response to hypoxia (Figure 1A), so was not investigated further. H3K4me3 and H3K36me3 are two of the most dynamic histone methylation modifications at the HTLV-1 provirus upon reactivation (M. Miura and A.K., unpublished data). However, their levels did not differ within our limits of detection in PBMCs cultured under hypoxia or normoxia.

### HTLV-1 Plus-Strand Gene Transcription Is Hypoxia Dependent but HIF Independent

**Figure 2:**
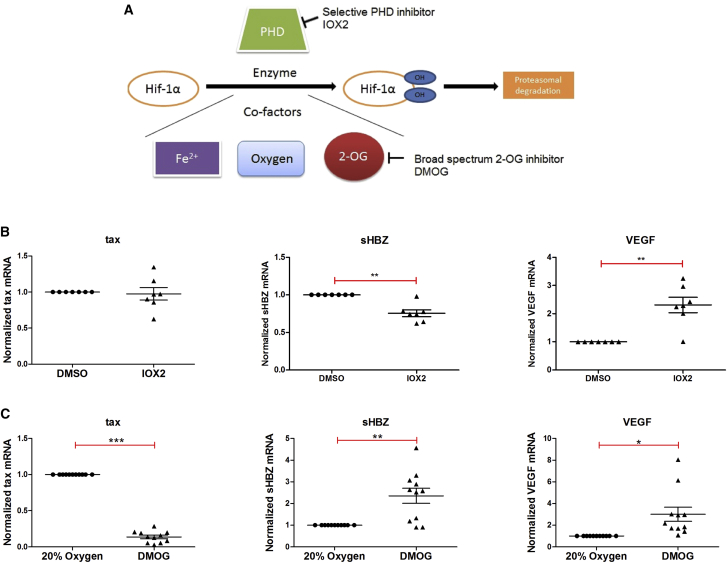
Hypoxia Mimics and HIF, and Their Role in Regulating HTLV-1 Transcription (A) Specificity of the two hypoxia mimics employed. IOX2 is a selective PHD inhibitor while DMOG is a competitive antagonist of 2-oxoglutarate (2-OG) binding, which acts as broad-spectrum inhibitor of 2OG-dependent oxygenases. (B and C) Evidence that HTLV-1 transcription is hypoxia-dependent, but HIF-independent. PBMCs isolated from HTLV-1-infected individuals were incubated overnight in the presence or absence of either IOX2 (B) or DMOG (C). RNA was extracted and subjected to qRT-PCR with primers specific for *tax* mRNA (plus strand), *sHBZ* mRNA (minus strand), or *VEGF* mRNA (positive control). Error bars represent the SE of samples treated respectively with IOX2 (n = 7) and DMOG (n = 11). Statistical significance was calculated using the two-tailed Student's t test (*p < 0.05, **p < 0.005, ***p < 0.0005).

Many transcriptional responses to hypoxia in cells are mediated by the α,β-HIF transcription factors (Kaelin and Ratcliffe, 2008). Under normoxic conditions (oxygen >5%), HIF-1α subunits are efficiently hydroxylated by the HIF prolyl hydroxylases (PHDs) and are rapidly degraded by the ubiquitin-proteasome system. The three human PHDs belong to the Fe(II) and 2-oxoglutarate (2-OG)-dependent oxygenase enzyme family, whose activities are dependent on molecular oxygen (O_2_). Under hypoxic conditions, PHD activities are inhibited and α,β-HIF is stabilized, thus enabling it to orchestrate the cellular response to hypoxia through its role as a transcriptional activator (Figure 2A) (Schofield and Ratcliffe, 2004). Because experimental work under hypoxic conditions imposes significant constraints, hypoxia mimics and HIF stabilizers have been developed to study hypoxia responses under normoxic conditions. Dimethyloxalylglycine (DMOG), a competitive antagonist of 2-OG, is a broad-spectrum inhibitor of 2-OG oxygenases (Figure 2A) (Jaakkola et al., 2001). In contrast, the recently developed molecule IOX2 is a much more selective inhibitor of the PHDs and stabilizes HIF without affecting the activities of most other 2-OG oxygenases (Figure 2A) (Chowdhury et al., 2013).

We studied the effects of DMOG and IOX2 on the transcriptional activity of the HTLV-1 provirus in PBMCs isolated from infected individuals. The PHD-selective inhibitor IOX2 had no effect on HTLV-1 plus-strand transcription (Figure 2B). The PHD inhibition activity of IOX2 in cells was confirmed by a significant increase in transcription of the HIF-1α-inducible gene *VEGF* (Figure 2B). Thus, the HIF-mediated transcriptional response probably does not play a direct role in the hypoxia-induced increase in HTLV-1 transcription. We observed a small but significant decrease in minus-strand *sHBZ* transcription on treatment with IOX2 (Figure 2B); the reasons for this effect are unknown, but could relate to altered levels of one of the many HIF target genes (which include chromatin-associated proteins [Pollard et al., 2008]). These results imply that HTLV-1 transcription is not directly HIF regulated, but does involve a hypoxia-related mechanism.

Compared with control cells, DMOG-treated infected PBMCs also showed a significant upregulation in positive control *VEGF* transcription, signifying HIF stabilization in DMOG-treated cells (Figure 2C) and consistent with the use of DMOG as an experimental mimic of hypoxia (Mole et al., 2003). However, DMOG exerted a strong and paradoxical effect on plus-strand HTLV-1 transcription. In contrast to hypoxia, which induced plus-strand transcription (Figure 1A), DMOG caused a significant and strong inhibition of HTLV-1 plus-strand transcription (Figure 2C). There was also an associated significant increase in minus-strand *sHBZ* transcription after DMOG treatment (Figure 2C). To rule out a possible effect of inhibitor cytotoxicity, we carried out dose-response analyses for both DMOG and IOX2. The results (Figure S2) confirmed the observations obtained above.

### The Opposing Effects of DMOG on Plus- and Minus-Strand HTLV-1 Transcription Are Independent of Each Other

**Figure 3:**
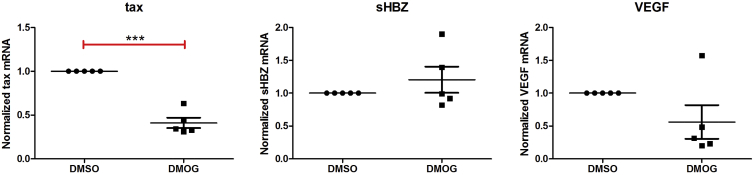
The Effect of DMOG on Plus-Strand HTLV-1 Transcription Is Independent of Its Effect on the Minus Strand HTLV-1-infected primary PBMCs were treated for 2 hr with either DMOG or DMSO (control). RNA was extracted and subjected to qRT-PCR with primers specific for *tax* mRNA (plus strand), *sHBZ* mRNA (minus strand), or *VEGF* mRNA (positive control). Error bars represent the SEM (n = 5). Statistical significance was calculated using the two-tailed Student's t test (***p < 0.0005).

The HTLV-1 Tax-mediated positive feedback loop governs plus-strand HTLV-1 transcription. Tax transactivates transcription of the plus-strand genes, including itself. HBZ is encoded by the minus strand and has been reported to inhibit Tax-mediated plus-strand transcription (Basbous et al., 2003, Yoshida et al., 2008). To analyze further the opposing effects of DMOG on plus- and minus-strand HTLV-1 transcription, we investigated the impact of DMOG on HTLV-1 transcription at an earlier time point. After 2 hr of culture of HTLV-1-infected patients' PBMCs with either DMOG or DMSO (control), there was a strong inhibition of *tax* mRNA transcription with DMOG treatment when compared with control (Figure 3). However, there was no corresponding significant increase in *sHBZ* transcription at 2 hr, suggesting that the inhibitory effect of DMOG on the plus strand is independent of its effect on the minus strand (Figure 3). However, a direct or indirect involvement of DMOG on the HBZ protein pools at the 2-hr time point cannot be ruled out. Also, there was no increase in transcription of the HIF-inducible gene *VEGF* at 2 hr, suggesting that the stabilization of HIF-1α in response to DMOG treatment did not occur at this early time point (Figure 3) in primary PBMCs.

### 2-OG Oxygenases Are Not Involved in Mediating Plus-Strand HTLV-1 Transcription

The 2-OG oxygenase enzyme family plays diverse roles in humans, including roles in collagen biosynthesis, fatty acid metabolism, hypoxic signaling, and nucleic acid and DNA repair and demethylation (Loenarz and Schofield, 2008). We wished to investigate whether specific 2-OG oxygenases regulated the observed epigenetic changes associated with HTLV-1 transcription in primary infected patients' PBMCs. The 2-OG oxygenases with important transcriptional regulatory roles in host cells include the HIF PHDs, Jumonji C histone lysine demethylases (JmjC KDMs), DNA cytosine hydroxylases (TET oxygenases), and the AlkB homolog nucleic acid demethylases (Loenarz and Schofield, 2008, Rose et al., 2011), some, but not all, of which are HIF target genes (Pollard et al., 2008).

We had already ruled out the direct involvement of PHDs by employing the selective PHD inhibitor IOX2, which had no effect on plus-strand HTLV-1 transcription (Figure 2B). To further investigate these results, we employed Methylstat (Luo et al., 2011) and JIB-04 (Wang et al., 2013), which are broad-spectrum JmjC KDM inhibitors (Figure S3A). These inhibitors had no significant effects on HTLV-1 plus-strand transcription, consistent with the conclusion that the JmjC KDMs do not play a direct regulatory role in HTLV-1 plus-strand transcription. In agreement with a previous report, Methylstat treatment resulted in a significant reduction in *VEGF* mRNA transcription (Cho et al., 2014). In contrast, JIB-04 treatment significantly induced *VEGF* transcription (Figure S3A). To our knowledge, there have been no prior studies examining the effect of JIB-04 on *VEGF* mRNA and angiogenesis. Furthermore, ChIP analysis showed no significant difference in either H3K4me3 or H3K36me3 when compared with control (DMSO) in the 5′-LTR and the *gag* region of the provirus (Figures S3B and S3C). There was a significant increase in H3K4me3 at the 3′-LTR in DMOG-treated samples compared with control (Figure S3B). This increase is consistent with the observed increase in *sHBZ* mRNA levels following DMOG treatment (Figure 2C). There was no difference in the DNA methylation profile between DMOG-treated and DMSO (control)-treated samples (Figure S3D), indicating that members of the TET and (at least some of the) AlkB homolog subfamilies of DNA demethylases/hydroxylases (nucleic acid oxygenases) are unlikely to be directly involved in regulating the observed spontaneous HTLV-1 plus-strand transcription. We conclude that the epigenetic effector 2-OG oxygenases are not directly involved in HTLV-1 plus-strand transcription (within our limits of detection).

### Metabolic Effects of DMOG on PBMCs from HTLV-1-Infected Individuals

2-OG (or α-ketoglutarate), in addition to being a co-substrate for 2-OG oxygenases, is also an important intermediate in metabolism, specifically the tricarboxylic acid (TCA) cycle, oxidative phosphorylation, and amino acid metabolism. To test the hypothesis that DMOG influences HTLV-1 transactivation through perturbation of these cellular metabolic pathways (in an HIF-independent manner), we treated HTLV-1-infected patients' PBMCs overnight with either 0.5 mM DMOG in DMSO, or DMSO alone (control). The cells were lysed and the extracts subjected to ion-exchange liquid chromatography coupled directly to tandem mass spectrometry (LC-MS/MS), to identify the metabolic pathways modulated by DMOG. Untargeted metabolite profiling measured 4,261 molecular species with a unique mass-to-charge ratio. The identified metabolites (n = 137) were sorted according to the maximum fold change in the normalized abundance between DMSO-treated and DMOG-treated samples, respectively. Any change that was statistically significant (p < 0.05) and exceeded 1.3-fold was analyzed further (raw data with identified metabolites are provided in File S2). N-Oxalylglycine (NOG) was found in high abundance in DMOG-treated cells but not in DMSO controls (File S2). This observation is commensurate with hydrolysis of the prodrug DMOG to NOG in cells by carboxylesterases (Zhdanov et al., 2015). Endogenous metabolites that showed a statistically significant difference were sorted according to the metabolic pathways in which they participate.

**Figure 4:**
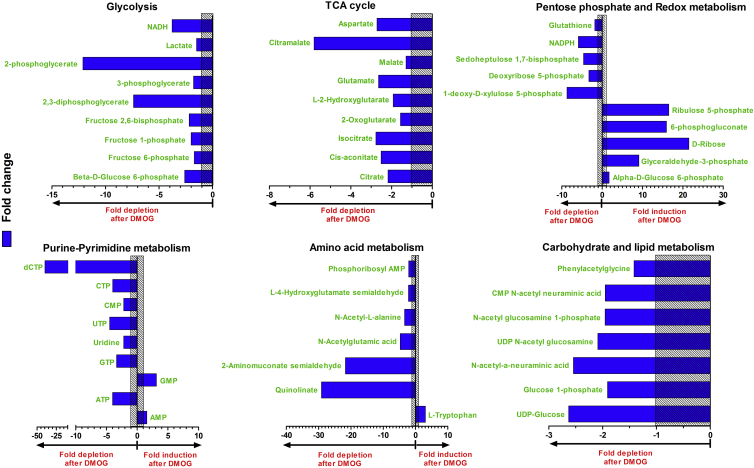
Metabolomic (LC-MS/MS) Analysis of DMOG-Treated HTLV-1-Infected Cells Primary PBMCs from HTLV-1-infected subjects (n = 5) were treated with either 0.5 mM DMOG or DMSO (control) overnight. Cells were lysed and subjected to metabolomic analysis by MS as mentioned in STAR Methods. The blue bars depict significant (p < 0.05) fold changes in the abundance of identified metabolites in response to DMOG treatment when compared with control samples. The bars on the right of the y axis correspond to metabolites induced by DMOG treatment while those on the left correspond to metabolites depleted after DMOG treatment. The metabolites have been grouped according to their respective metabolic pathways. The shaded area corresponds to a fold change from −1 to 1 (no change in metabolite levels in response to DMOG treatment).
(2) TCA cycle. Being an analog of the TCA cycle metabolite 2-OG, DMOG affects the TCA cycle (Rendina et al., 2013); Specifically, the levels of the TCA cycle intermediates citrate, *cis*-aconitate, isocitrate, 2-OG, and malate were significantly lower in DMOG-treated HTLV-1-infected PBMCs than in control (DMSO-treated) cells. Only succinate levels remained unchanged among the measured TCA-cycle metabolites in response to DMOG treatment. DMOG inhibits mitochondrial respiration independently of its hypoxia mimic effect (Zhdanov et al., 2015).
(3) Pentose phosphate pathway. Higher levels of glyceraldehyde-3-phosphate, α-D-glucose 6-phosphate, 6-phosphogluconate, ribulose 5-phosphate, and D-ribose in combination with low levels of 1-deoxy-D-xylulose 5-phosphate, deoxyribose 5-phosphate, and sedoheptulose 1,7-bisphosphate in DMOG-treated samples when compared with control pointed toward a downstream inhibition in the pentose phosphate pathway in response to DMOG treatment.
(4) Redox metabolism. The pentose phosphate pathway is responsible for synthesis of NADPH, a reducing equivalent in cell metabolism (Stincone et al., 2015), e.g., in the conversion of oxidized glutathione disulfide (GSSG) to reduced glutathione (GSH) for counteracting cellular redox stress. The decrease in cellular NADPH levels, together with the associated decrease in glutathione levels in DMOG-treated cells, suggests that the DMOG-treated cells were under oxidative stress. This proposal is supported by the observed impairment (in response to DMOG) in oxidative phosphorylation ((2) above) and the mitochondrial electron transport chain, both of which generate oxidative stress (Cadenas and Davies, 2000, Rendina et al., 2013).
(5) Purine and pyrimidine metabolism. While levels of purine metabolites adenosine and guanosine monophosphate (AMP and GMP) were significantly higher in DMOG-treated cells when compared with controls, the corresponding nucleoside triphosphates (ATP and GTP) were significantly depleted. All pyrimidine metabolites measured (UTP, UMP, CTP, and CMP) were depleted in DMOG-treated cells when compared with control. dCTP, a precursor for DNA synthesis, was strongly inhibited by DMOG treatment.
(6) Amino acid, carbohydrate, and lipid metabolism. DMOG treatment resulted in a significant increase in L-tryptophan levels. This might be expected given the strong concomitant reduction in tryptophan degradation products, quinolinic acid and 2-aminomuconic acid semialdehyde, observed in the DMOG-treated samples. Inhibition of glucose metabolism by DMOG might in turn inhibit many anabolic processes, because of the reduction in ATP and NADH. The observed significant decrease in the levels of N-acetylated metabolites could be due to the limited availability of acetyl-coenzyme A resulting from an inhibition of glycolysis and the TCA cycle.

The results showed significant changes in six metabolic pathways:

(1) Glycolysis. A significant reduction was observed in the levels of most of the measured glycolytic intermediates in response to DMOG treatment. In particular, intermediates toward the end of glycolysis: 2,3-diphosphoglycerate and 3-phosphoglycerate were highly depleted in DMOG-treated cells (Figure 4). Glyceraldehyde-3-phosphate and α-D-glucose 6-phosphate were the only glycolytic intermediates that showed significantly higher levels in DMOG-treated cells compared with control (Figure 4). Pyruvate levels remained largely unchanged in response to DMOG treatment (File S2). There was a significant reduction in the level of glycolysis and TCA-cycle products NADH and ATP in response to DMOG treatment.

Thus most of the observed metabolic effects of DMOG, discussed above, are secondary to its perturbation of glycolysis, the TCA cycle, and oxidative phosphorylation (Figure 4). Hence we went on to target these pathways with specific inhibitors to study whether selective inhibition of certain enzymes could inhibit *tax* transcription in HTLV-1-infected primary PBMCs.

### The Rate of Glycolysis, but Not the Concentrations of TCA-Cycle Intermediates, Is Linked to Plus-Strand HTLV-1 Transcription

**Figure 5:**
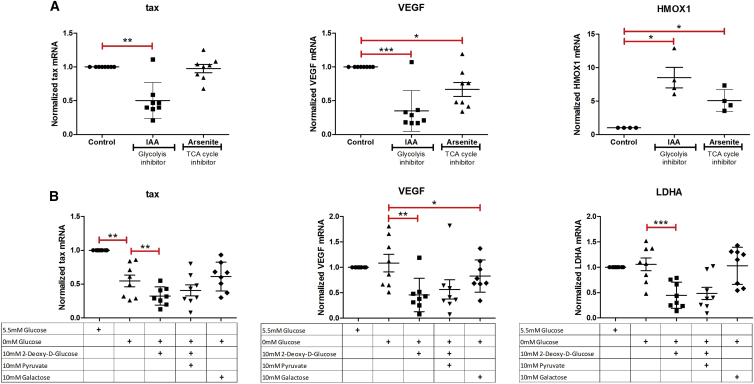
The Rate of Glycolysis, but Not the TCA Cycle, Is Intimately Linked to Plus-Strand HTLV-1 Transcription HTLV-1-infected primary PBMCs were treated overnight with either control (water), iodoacetic acid (IAA), or sodium arsenite (A) or cultured under indicated concentrations of glucose-containing RPMI in the presence or absence of the glycolysis inhibitor 2-deoxy-D-glucose (2-DG) and TCA-cycle inducers sodium pyruvate and galactose (B). RNA was extracted and subjected to qRT-PCR with primers specific for *tax* mRNA (plus strand), heme oxygenase 1 (*HMOX1*) mRNA (positive control for IAA and arsenite), *VEGF* mRNA, and lactate dehydrogenase A (*LDHA*) mRNA (indicator for rate of glycolysis). Error bars represent the SEM (n = 8, except for HMOX1 in A where n = 4). Statistical significance was calculated using the two-tailed Student's t test (***p < 0.0005, **p < 0.005, *p < 0.05).

To investigate the influence of glucose metabolism on HTLV-1 transcription, we tested the effects of the TCA-cycle inhibitor sodium arsenite (Bergquist et al., 2009) and the glycolysis inhibitor iodoacetate (IAA) (Sabri and Ochs, 1971) (Figure 5A) and quantified their effects on HTLV-1 plus-strand transcription in primary PBMCs derived from HTLV-1-infected individuals.

IAA treatment significantly inhibited HTLV-1 *tax* mRNA transcription, whereas arsenite caused no change in *tax* mRNA levels at the concentration tested (Figure 5A). The stress-inducible enzyme heme oxygenase 1 (HMOX1) served as the positive control for the biological activity of the drugs (Figure 5A). IAA and arsenite also inhibited cellular *VEGF* mRNA levels (Figure 5A). These results suggest that glucose metabolism influences reactivation of HTLV-1 from latency and reinforce the conclusion that HTLV-1 *tax* mRNA transcription is independent of HIF and intracellular oxidative stress. Neither IAA nor arsenite made an impact on minus-strand HTLV-1 transcription (Figure S4A).

As a further test of the involvement of glucose metabolism in HTLV-1 transactivation from latency, primary PBMCs from HTLV-1-infected individuals were cultured in RPMI medium with either 5.5 mM glucose with 10% fetal bovine serum (FBS) (physiological glucose concentrations) or 0 mM glucose with 10% FBS in the presence or absence of the glycolysis inhibitor 2-deoxy-D-glucose (2-DG) (Zhong et al., 2009) and the TCA-cycle inducers sodium pyruvate (Diers et al., 2012) or galactose (Aguer et al., 2011) (Figure 5B).

PBMCs from HTLV-1-infected individuals cultured overnight in RPMI with 0 mM glucose +10% FBS expressed significantly lower levels of HTLV-1 *tax* mRNA than did PBMCs cultured in RPMI with physiological (5.5 mM) glucose +10% FBS. Addition of the glycolysis inhibitor 2-DG to the 0-mM glucose medium led to a further significant suppression of HTLV-1 plus-strand transcription. Further addition of the TCA-cycle inducer sodium pyruvate (0 mM glucose + 2-DG + pyruvate) did not rescue the inhibitory effect of 2-DG on HTLV-1 *tax* transcription. These observations are consistent with the data shown in Figure 5A; they indicate that glycolysis, but not the TCA cycle, plays an important role in HTLV-1 plus-strand transcription. Galactose, an inducer of the TCA cycle and oxidative phosphorylation, had no effect on HTLV-1 plus-strand transcription when added to 0-mM glucose medium, strengthening the conclusion that the TCA cycle is not involved in HTLV-1 reactivation from latency. Like IAA and arsenite, 2-DG treatment also resulted in a significant reduction in cellular *VEGF* mRNA levels. Inhibition of glycolysis by 2-DG also resulted in a significant decrease in the mRNA of lactate dehydrogenase A, a surrogate marker of the rate of glycolysis (Figure 5B). There was no significant difference in *sHBZ* transcription upon either inhibition of glycolysis (by incubation in 0 mM glucose or 2-DG) or TCA-cycle induction (by pyruvate or galactose) when compared with control (Figure S4B).

### Inhibition of the Mitochondrial Electron Transport Chain Inhibits Plus-Strand HTLV-1 Transcription

**Figure 6:**
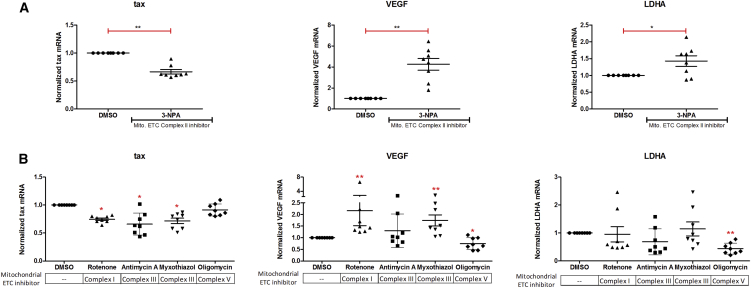
Inhibition of the Mitochondrial Electron Transport Chain Inhibits Plus-Strand HTLV-1 Transcription HTLV-1 infected primary PBMCs were cultured overnight in the presence of the indicated compounds in (A) and (B). RNA was extracted and subjected to qRT-PCR with primers specific for *tax* mRNA (plus strand), *VEGF* mRNA, and *LDHA* mRNA. Error bars represent the SEM (n = 8). Statistical significance was calculated by the paired Wilcoxon signed-rank test (**p < 0.005, *p < 0.05).

Next, we studied the effect of inhibition of the mitochondrial electron transport chain (ETC) on HTLV-1 plus-strand transcription. PBMCs from HTLV-1-infected individuals were incubated overnight in the presence or absence of inhibitors of mitochondrial ETC complex II (3-nitropropionic acid: Figure 6A), complex I (rotenone: Figure 6B), complex III (antimycin A and myxothiazol: Figure 6B), and complex V (oligomycin: Figure 6B) (Rohlena et al., 2013).

Four out of five mitochondrial ETC complex inhibitors tested caused a significant reduction in plus-strand HTLV-1 transcription (Figures 6A and 6B): only oligomycin, an inhibitor of complex V, had no impact on HTLV-1 *tax* transcription (Figure 6B). These inhibitors had varying effects on *VEGF* and *LDHA* mRNA levels (Figures 6A and 6B). Also, there was no change in minus-strand *sHBZ* transcription in response to ETC-inhibitor treatment (Figure S4C). These results suggest that the mode of action of the ETC inhibitors on plus-strand HTLV-1 transcription is an independent phenomenon and is not mediated through their perturbation of the glycolytic and hypoxia pathways in cells.

## Discussion

CD4^+^ T lymphocytes, the primary reservoir of HTLV-1 in humans, are routinely exposed *in vivo* to alterations in the microenvironment such as changes in the oxygen tension or the concentration of glucose and other nutrients. We hypothesized that such changes influence the transcriptional activity of the HTLV-1 provirus, which is usually silent in freshly isolated PBMCs but undergoes strong spontaneous activation *ex vivo*. To minimize artifacts due to *in vitro* selection, we studied fresh PBMCs isolated from HTLV-1-infected individuals.

HTLV-1-infected primary PBMCs cultured under conditions of physiological hypoxia (1% or 2% oxygen) showed a significant enhancement in HTLV-1 plus-strand transcription when compared with those cultured under ambient oxygen conditions (∼20% oxygen) (Figure 1). Hypoxia has been shown to have variable effects on viral expression within cells. For example, hypoxia induces lytic replication in human herpesvirus 8 (Davis et al., 2001), whereas Tat-induced HIV-1 transcription is inhibited by hypoxia (Charles et al., 2009). Thus, viruses have developed different adaptive responses to varying oxygen levels within the body. By employing a specific HIF stabilizer, IOX2 (Chowdhury et al., 2013), we showed that the hypoxia-mediated effect on HTLV-1 transcription appeared to be independent of HIF (Figure 2B). The effect of physiological hypoxia on HTLV-1 transcription suggests that the dynamics of HTLV-1 reactivation that we observe in freshly isolated PBMCs from venous blood differ from the dynamics in other compartments such as solid lymphoid tissue or bone marrow, where the oxygen tension is typically 1%–2% (Ivanovic, 2009, Ohta et al., 2011). Since hypoxia enhances plus-strand proviral transcription, the infected cells in these compartments might be more likely to support productive viral replication and spread. Consistent with this hypothesis, Yasunaga et al. (2016) reported higher levels of *tax* mRNA in the bone marrow than in other tissues.

In contrast to the effect of hypoxia, the hypoxia mimic DMOG potently inhibited proviral plus-strand transcription in HTLV-1-infected primary PBMCs (Figure 2C). We therefore investigated the HIF-independent effects of DMOG, either as an inhibitor of 2-OG oxygenases or as an inhibitor of glucose metabolism and the TCA cycle. Having ruled out the involvement of epigenetic effector 2-OG oxygenases including PHDs (Figures 2B and 2C), JmjC lysine demethylases (Figures S3A–S3C), and nucleic acid oxygenases (Figure S3D), we studied the influence of glucose metabolism on HTLV-1 transcription. Mass spectrometry of DMOG-treated HTLV-1-infected primary PBMCs revealed that DMOG significantly inhibited all the metabolic pathways closely linked to glucose metabolism (Figure 4). It is known that DMOG inhibits the TCA cycle and mitochondrial respiration (Rendina et al., 2013, Zhdanov et al., 2015), but direct inhibition of glycolysis by DMOG has not previously been reported.

Chemical inhibition of glycolysis by either iodoacetic acid or 2-DG significantly reduced plus-strand HTLV-1 transcription (Figure 5). However, neither an inhibitor (arsenite) nor inducers (sodium pyruvate and galactose) of the TCA cycle altered HTLV-1 transcription. We conclude that glycolysis regulates reactivation from latency of the integrated HTLV-1 provirus. Consistent with this effect, we saw a significant reduction in HTLV-1 plus-strand transcription in PBMCs cultured in glucose-free medium when compared with those cultured under physiological glucose concentration (5.5 mM). The glucose receptor GLUT-1 is a cellular receptor for HTLV-1 infection (Manel et al., 2003); expression of GLUT-1 is induced by hypoxia and other forms of cellular stress.

Four out of five inhibitors of the mitochondrial ETC tested significantly reduced HTLV-1 transcription (Figure 6). Specifically, inhibitors of ETC complexes involved in electron transfer (complexes I, II, and III) reduced HTLV-1 plus-strand transcription. However, inhibition of mitochondrial ATP synthase (complex V) by oligomycin had no impact (Figure 6). It is unclear why oligomycin had no effect on HTLV-1 transcription at the tested concentration. The ETC inhibitors had variable effects on expression of the hypoxia-inducible gene *VEGF* and the glycolytic enzyme *LDHA*, suggesting that their effect on HTLV-1 transcription is independent of hypoxia and glycolysis. Ciminale and colleagues have shown that the p13 accessory protein of HTLV-1 modulates mitochondrial membrane potential by facilitating an inward K^+^ current, thereby increasing ETC activity and reactive oxygen species production, consistent with a link between mitochondrial function and the HTLV-1 life cycle (Silic-Benussi et al., 2009, Silic-Benussi et al., 2010).

We propose that the strong inhibition of HTLV-1 caused by DMOG is due to inhibition of both glycolysis and the mitochondrial ETC. Thus, glycolysis and the mitochondrial ETC play an important role in regulating HTLV-1 plus-strand transcription, whereas the TCA cycle does not play a direct role. In the light of the recent observations that different subsets of T cells rely on different metabolic pathways for their energy needs (Buck et al., 2015, Dimeloe et al., 2017), it is plausible that the TCA cycle contributes less than glycolysis to the metabolism of HTLV-1-infected CD4^+^ T cells.

A typical HTLV-1-infected individual has approximately 10^4^ to 10^5^ different HTLV-1-infected T cell clones, each with a unique proviral integration site (Gillet et al., 2011, Laydon et al., 2014, Melamed et al., 2013). We conclude that both viral determinants (e.g., genomic integration site, orientation) and the host microenvironment (e.g., glucose concentration, oxygen levels) determine the likelihood of spontaneous reactivation of an integrated HTLV-1 provirus from a latent state.

## Significance

**Retroviruses such as HTLV-1 persist life-long in their host by integrating a copy of their genetic material into the host cellular genomic DNA. Although HTLV-1 infection is asymptomatic in most cases, in a subset of infected individuals (∼10%) it causes an aggressive hematological malignancy or a debilitating neuroinflammatory condition. Infection is considered largely latent due to the absence of viral RNA and proteins in fresh blood samples. However, when blood obtained from HTLV-1-infected individuals is cultured *ex vivo*, there is a spontaneous increase in plus-strand HTLV-1 transcription, which suggests that changes in the extracellular microenvironment play an important deterministic role in viral expression. Here, we identify two factors in the microenvironment that regulate HTLV-1 proviral latency and expression. First, we show that physiological hypoxia (1% or 2% oxygen), as present in the lymphoid organs and bone marrow, enhances HTLV-1 transcription. Second, inhibition of glycolysis or the mitochondrial electron transport chain suppresses plus-strand HTLV-1 transcription. We conclude that both glucose metabolism and oxygen availability regulate HTLV-1 transcription. The significance of these results is twofold. First, the identification of two microenvironmental factors that regulate HTLV-1 expression constitutes a basic advance in the understanding of HTLV-1 persistence and pathogenesis. Second, targeting these pathways with currently available as well as novel therapies could complement existing antiretrovirals and improve treatment efficiency.**

## STAR★Methods

### Key Resources Table

**Table:** 

REAGENT or RESOURCE	SOURCE	IDENTIFIER
**Antibodies**		

ChIPAb+Trimethyl-Histone H3 Lys 4	Millipore	Cat# 17-614 (RRID: AB_11212770)
ChIPAb+Trimethyl-Histone H3 Lys 36	Millipore	Cat# 17-10032 (RRID: AB_10615601)
ChIPAb+ Trimethyl-Histone H3 Lys 27	Millipore	Cat# 17-622 (RRID: AB_916347)

**Biological Samples**		

Human patient samples provided as excel File S1	NCHR	http://www.htlv1.eu/

**Chemicals, Peptides, and Recombinant Proteins**		

List of chemicals used provided in Table S1	Refer to Table S1	Refer to Table S1

**Critical Commercial Assays**		

Methyl Collector Ultra kit	Active Motif	Cat# 55005

**Oligonucleotides**		

List of oligonucleotides and probes used provided as Table S2	Refer to Table S2	Refer to Table S2

**Software and Algorithms**		

LinRegPCR- qPCR analysis software	HFRC, Netherlands	http://www.gene-quantification.com/LinRegPCR_help_manual_v11.0.pdf
Progenesis QI	Waters, U.K.	http://www.waters.com/waters/en_US/Progenesis-QI-Software/nav.htm?cid=134790655&locale=en_US

### Contact for Reagent and Resource Sharing

Further information and requests for reagents may be directed to, and will be fulfilled by, the corresponding author Charles R.M. Bangham ( c.bangham@imperial.ac.uk )

### Experimental Model Details

#### Human Specimens

A list of patient blood samples tested has been provided in File S1. All donors attended the National Centre for Human Retrovirology (NCHR) at Imperial College Healthcare NHS Trust, St Mary's Hospital, London and gave written informed consent in accordance with the Declaration of Helsinki to donate blood samples to the Communicable Diseases Research Tissue Bank which is approved by the UK National Research Ethics Service (15/SC/0089).

### Method Details

#### Primary T-Cell Isolation and Culture

Venous blood samples from HTLV-1-infected individuals attending the NCHR HTLV clinic were separated using Histopaque and PBMCs stored in liquid nitrogen. CD8+ T-cells were depleted from PBMCs with Dynabeads CD8 (ThermoFisher Scientific), using the manufacturer’s instructions. Equal volumes of RPMI with L-Glutamine without Glucose (BE12-752F, Lonza) and RPMI with L-Glutamine and 11mM Glucose (BE12-702F) were mixed to make RPMI with L-Glutamine and 5.5mM Glucose. This medium was used for cell culture with 10% FBS (Gibco) in all experiments, unless otherwise stated.

#### Hypoxia Culture

A ‘Hypoxylab’ (Oxford Optronix, Oxford, UK) - hypoxia workstation and incubator was employed for all hypoxia-related experiments. All media and consumables used in hypoxic culture were conditioned to the target oxygen concentration (1% or 2%) before use. Primary PBMCs were manipulated and cultured in the HypoxyLab at the desired oxygen concentration for the indicated time. *In situ* dissolved oxygen in the culture media was measured using the integrated OxyLite™ (Oxford Optronix) oxygen sensor to accurately monitor oxygen availability in the cellular microenvironment.

#### Inhibitors

Multiple chemical inhibitors of different cellular pathways were employed in this study. A comprehensive list of all the compounds used along with the corresponding references is provided in Table S1. Stocks of each inhibitor were prepared in either DMSO or water, and diluted to their desired concentration in culture medium to study their effects on HTLV-1 transcription.

#### RNA Extraction and qRT-PCR

RNA was extracted from cultured PBMCs using the RNeasy Plus Mini kit (Qiagen). cDNA was synthesised from the extracted RNA using the Transcriptor First Strand cDNA Synthesis kit (Roche) by following the manufacturer instructions. An additional no-RT control was included for each cDNA sample synthesised. The kinetic PCR amplification was carried out using the Viia7 Real-time PCR system (Applied Biosystems) with gene-specific primers and Fast SYBR Green Master Mix. The list of primers used is given in Table S2.

#### Chromatin Immunoprecipitation (ChIP)-qPCR

Up to 10 million cells were crosslinked with 1% formaldehyde for 10min at room temperature. Fixed cells were quenched with 125mM Glycine and lysed with Cell lysis buffer (5mM PIPES-KOH pH 8.0; 85mM KCl; 0.5% NP-40). Subsequently nuclei were pelleted by centrifugation at 2000rpm for 5 minutes and subjected to lysis with 130 μl Nuclear lysis buffer (1% SDS; 10mM EDTA; 50mM Tris-HCl pH8.0). Nuclear lysates were sonicated using the Covaris S220 sonicator in a microTUBE (Covaris), with the following Sonolab7.2 program parameters: Peak Incident Power: 105 Watts; Duty Factor: 10%; Cycles per burst: 200; Treatment time: 480 sec; Distilled Water Temperature: 4-6°C. Sonicated lysates were subjected to immunoprecipitation (IP) using the following antibodies: ChIPAb+Trimethyl-Histone H3 Lys 4 (Millipore, 17-614) and ChIPAb+Trimethyl-Histone H3 Lys 36 (Millipore, 17-10032), ChIPAb+ Trimethyl-Histone H3 Lys 27 (Millipore, 17-622) and corresponding control IgG overnight at 4°C in the presence of MagnaChIP A+G magnetic beads (Millipore). A 10% IP input sample was collected separately as a reference for relative quantification. The bead-bound immunoprecipitated DNA was washed sequentially for 10 minutes each at 4°C with the following wash buffers- Low salt wash buffer (0.1% SDS; 1% Triton X-100; 2mM EDTA; 20mM Tris-HCl pH8.0; 150mM NaCl), High salt wash buffer (0.1% SDS; 1% Triton X-100; 2mM EDTA; 20mM Tris-HCl pH8.0; 500mM NaCl), LiCl wash buffer (0.25M LiCl; 1% NP-40; 1% Deoxycholic acid (sodium salt); 1mM EDTA; 10mM Tris-HCl pH8.0) and 2 washes with TE buffer (1mM EDTA; 10mM Tris-HCl pH8.0). The DNA was eluted from the beads by IP elution buffer (1% SDS; 0.1 M NaHCO3). The eluted DNA was reverse cross-linked at 65°C overnight in the presence of 300mM NaCl and thereafter subjected to proteinase K digestion at 45°C for 2 hours. The immunoprecipitated and input DNAs were purified by using the QIAquick PCR Purification Kit (Qiagen). The DNA enrichment in ChIP samples was quantified using region-specific primers for 5’-LTR junction, *Gag*, *Pol*, *Env*, *vCTCF*, *Tax* and 3’-LTR junction of the HTLV-1 provirus and corresponding qPCR TaqMan probes (sequences described in Table S2). The kinetic PCR amplification was carried out using the Viia7 Real-time PCR system (Applied Biosystems) with TaqMan Gene Expression Master Mix (ThermoFisher Scientific).

#### Methylated DNA Immunoprecipitation (MeDIP)

Genomic DNA from PBMCs was extracted using a QIAamp DNA Mini kit (Qiagen), following the manufacturer’s instructions. The extracted DNA was sonicated using a Covaris S220 machine (Sonolab 7.2 program: Peak Incident Power: 175 Watts; DutyFactor: 10%; Cycles per burst: 200; treatment time: 105 sec; Distilled Water Temperature: 5-8°C). MeDIP assays were carried out using the MethylCollector Ultra kit (Active Motif) according to the manufacturer’s protocol. Immunoprecipitated DNA was quantified by qPCR as described above for ChIP, using the same primers and probes.

#### Metabolomics Protocol

##### Sample Preparation

PBMCs isolated from HTLV-1 infected individuals were cultured overnight in the presence of 0.5mM DMOG or DMSO (control). Briefly, pelleted cells were lysed with ice-cold 80% methanol (MS grade). 0.2ml of each lysate was then filtered using a 10kd molecular weight cut-off filter (Amicon Ultra, Millipore). The liquid which had passed though the filter was then placed in an autosampler vial (Waters) and stored at -80°C. On the day of analysis the extract was allowed to warm to 4°C in the chilled autosampler and then analysed directly by LC/MS/MS.

##### LC/MS/MS

Metabolite analyses were performed using a Thermo Scientific ICS-5000+ ion chromatography system coupled directly to a Q-Exactive HF Hybrid Quadrupole-Orbitrap mass spectrometer with a HESI II electrospray ionisation source (Thermo Scientific, San Jose, CA). The ICS-5000+ HPLC system incorporated an electrolytic anion generator (KOH) which was programmed to produce an OH- gradient from 5-100mM over 37 minutes. An inline electrolytic suppressor removed the OH- ions and cations from the post-column eluent prior to eluent delivery to the electrospray ion source of the MS system (Thermo Scientific Dionex AERS 500). A 10 μL partial loop injection was used for all analyses and the chromatographic separation was performed using a Thermo Scientific Dionex IonPac AS11-HC 2 × 250 mm, 4 μm particle size column with a Dionex Ionpac AG11-HC 4 μm 2x50 guard column inline. The IC flow rate was 0.250 mL/min. The total run time was 37 minutes and the hydroxide ion gradient comprised as follows: 0mins, 0mM; 1min, 0mM; 15mins, 60mM; 25mins, 100mM; 30mins, 100mM; 30.1mins, 0mM; 37mins, 0mM. Analysis was performed in negative ion mode using ascan range from 80-900 and resolution set to 70,000. The tune file source parameters were set as follows: Sheath gas flow 60; Aux gas flow 20; Spray voltage 3.6; Capillary temperature 320; S-lens RF value 70; Heater temperature 450. AGC target was set to 1e6 and the Max IT value was 250ms. The column temperature was kept at 30°C throughout the experiment. Full scan data were acquired in, continuum mode across the mass range m/z 60-900.

### Quantification and Statistical Analysis

#### Processing and Statistical Analysis of Metabolomics Data

##### Data Processing

Raw data was processed using Progenesis QI for small molecules (Waters, Elstree, UK). Briefly this encompassed chromatographic peak alignment, isotope cluster recognition (peak picking) and compound identification. Identification of compounds in experimental samples was based on matching to an in-house library of authentic standards, using four measured parameters for each compound from the database. These were: accurate mass measurement (<5ppm) based on theoretical mass derived from the chemical formula, experimental retention time window of 0.5mins, isotope pattern recognition (calculated from chemical formula) and matching with fragmentation patterns from an authentic standard where these were available from survey scans. All values in the database were obtained from the analysis of authentic standard compounds.

##### Statistical Analysis

Statistical analysis was performed using Progenesis QI and the EZ info plugin for Progenesis QI developed by Umertrics. Supervised and unsupervised modelling of the data was performed (PCA and OPLS-DA). Volcano plots, S-plots and VIP values were extracted from OPLS-DA models to help identify highly significant compounds and potential biomarkers. p-values, %CV and fold-changes associated with the statistical comparison of experimental groups were calculated for each metabolite using progenesis QI. These data were used to identify and evaluate potential metabolic biomarkers. The identified metabolites were sorted according to the maximum fold difference in normalized abundance between DMSO-treated and DMOG-treated samples respectively. Any difference in metabolite abundance, which was statistically significant (p<0.05), and fold change >1.3-fold, was analysed further. The resultant metabolite list was mapped according to the metabolic pathways they were associated with.

#### Quantification of qRT-PCR Data

LinRegPCR-Ct method (Cikos et al., 2007) was used for relative quantification of target mRNA levels. LinRegPCR software was used to determine baselines, threshold and mean efficiency (E) of the reaction to calculate target mRNA quantity (R0), where R0 = Threshold/ E^Ct^. All values were normalized to their respective 18S rRNA levels, which was the internal PCR control.

#### Quantification of ChIP-qPCR and MeDIP Data

DNA enrichment was calculated as % Input = (E^ΔCt^)*10, where ΔCt= Ctinput- Ctsample. LinRegPCR software was used to determine the mean efficiency of the reaction for each primer pair.

#### Statistical Analyses

Two-tailed Student’s T test, Wilcoxon matched pairs signed rank test and 1-way ANOVA with post-test for linear trend were employed for statistical analysis of data as described in the corresponding figure legends.

### Data and Software Availability

The raw metabolomics data with the identified metabolites has been provided in File S2.

## Author Contributions

Conceived and designed the experiments: A.K., C.C.T., C.J.S., and C.R.M.B.; Performed the experiments: A.K., M.M., and J.S.M.; Analyzed the data: A.K., J.S.M., C.J.S., and C.R.M.B.; Contributed reagents/materials/analysis tools: C.C.T., C.J.S., and J.S.M.; Writing – original draft: A.K. and C.R.M.B.; Writing – review and editing: A.K., C.R.M.B., C.J.S., J.S.M., and G.P.T.; Recruited patients: G.P.T.

## Supplemental Information

Document S1. Figures S1–S4 and Tables S1 and S2

File S1. List of Patient Blood Samples Used in This Study, Related to Figures 1–6

File S2. List of Identified Metabolites in Response to DMSO (Control) or DMOG Treatment in Primary PBMCs Derived from HTLV-1 Infected Individuals, Related to Figure 4

Document S2. Article plus Supplemental Information
