# Supplementary material for: Glucose Metabolism and Oxygen Availability Govern Reactivation of the Latent Human Retrovirus HTLV-1
Source: Cell Chem Biol. 2017 Nov 16;24(11):1377–1387.e3. doi: 10.1016/j.chembiol.2017.08.016 (PMC5696563; doi:10.1016/j.chembiol.2017.08.016)
Supplement: Document S1. Figures S1–S4 and Tables S1 and S2 [file mmc1.pdf]

Cell Chemical Biology, Volume *24*

## Supplemental Information

# Glucose Metabolism and Oxygen Availability Govern Reactivation of the Latent Human Retrovirus HTLV-1

**Anurag Kulkarni, Manuel Mateus, Cyrille C. Thinnes, James S. McCullagh, Christopher J. Schofield, Graham P. Taylor, and Charles R.M. Bangham**

# Glucose metabolism and oxygen availability govern reactivation from latency of the human retrovirus HTLV-1

## Supplemental information

Supplementary figures S1 to S4

Supplementary tables S1 and S2

**Figure S1. Related to Figure 1**

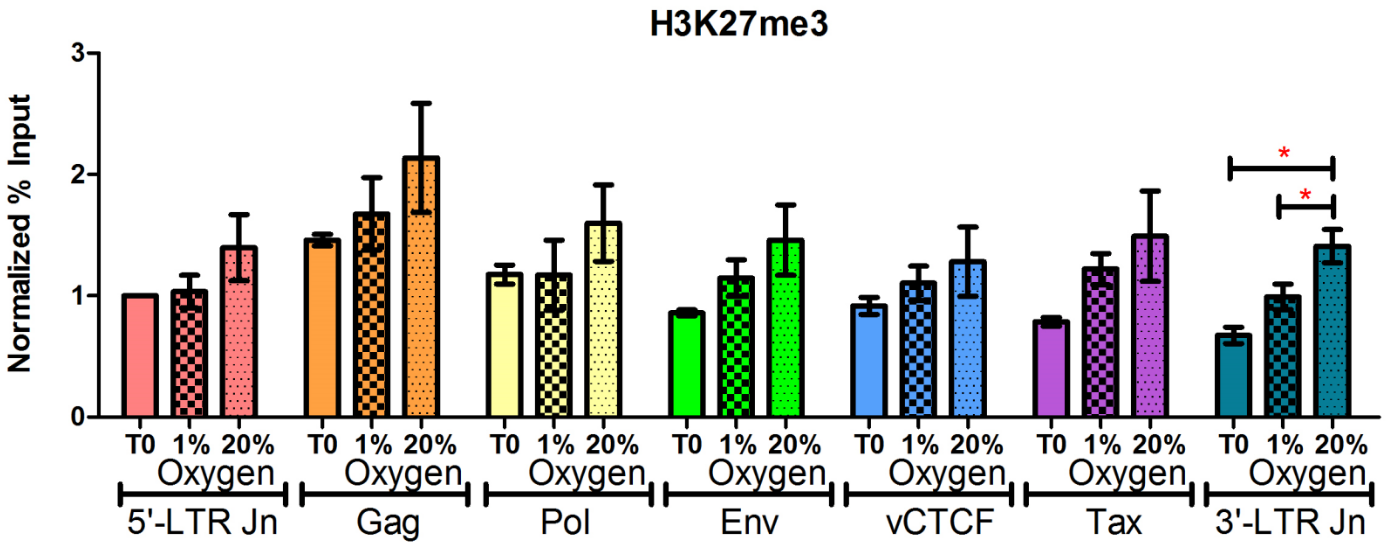


**Figure S1: H3K27me3 status changes in the HTLV-1 provirus upon reactivation under physiological (1%) hypoxia or normoxia.** HTLV-1-infected PBMCs were either fixed immediately (T0) or cultured under hypoxia (1% oxygen) or normoxia (20% oxygen) overnight and subsequently fixed and subjected to ChIP with antibodies directed against H3K27me3 and IgG, and primers specific for the 5'-LTR Jn, *Gag*, *Pol*, *Env*, *vCTCF*, *Tax* and 3'-LTR Jn of the HTLV-1 provirus. Enrichment is expressed as % input DNA and normalized to T0 at the 5'-LTR junction. Error bars represent the standard error of 4 independent ChIP experiments. Statistical significance was calculated using the two-tailed Student's T-test where * represents $p<0.05$.

**Figure S2. Related to Figure 2**

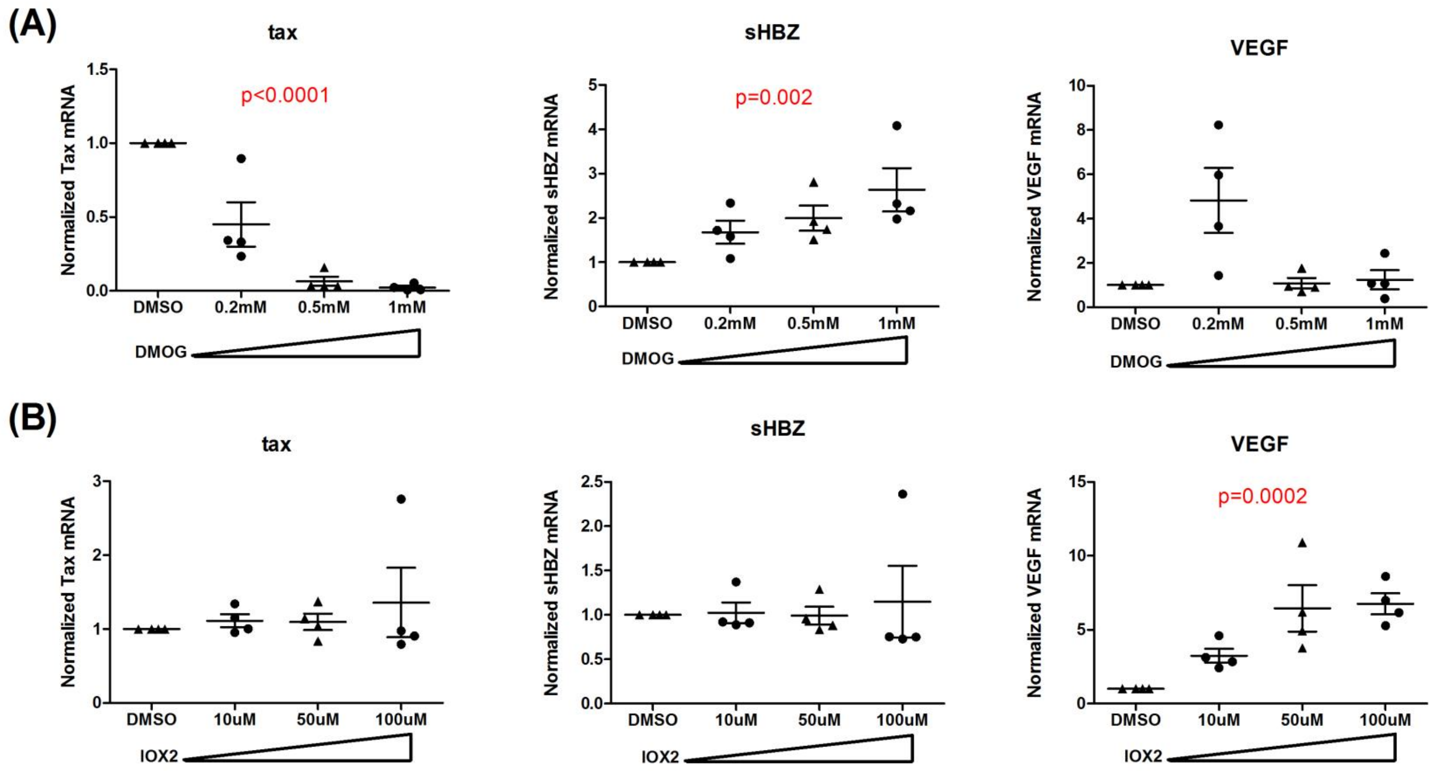


**Figure S2: Dose-response effects of DMOG and IOX2 on HTLV-1 transcription.** HTLV-1-infected primary PBMCs were treated overnight with increasing concentrations of DMOG **(A)** or IOX2 **(B)**. RNA was extracted and subjected to RT-qPCR with primers specific for *tax* mRNA (plus-strand), *sHBZ* (minus-strand) or *VEGF* mRNA (positive control). Error bars represent the standard error of the mean (n=4). Statistical significance was calculated using 1-way ANOVA test with post-test for linear trend. P-values < 0.05 are significant.

**Figure S3. Related to Figure 2**

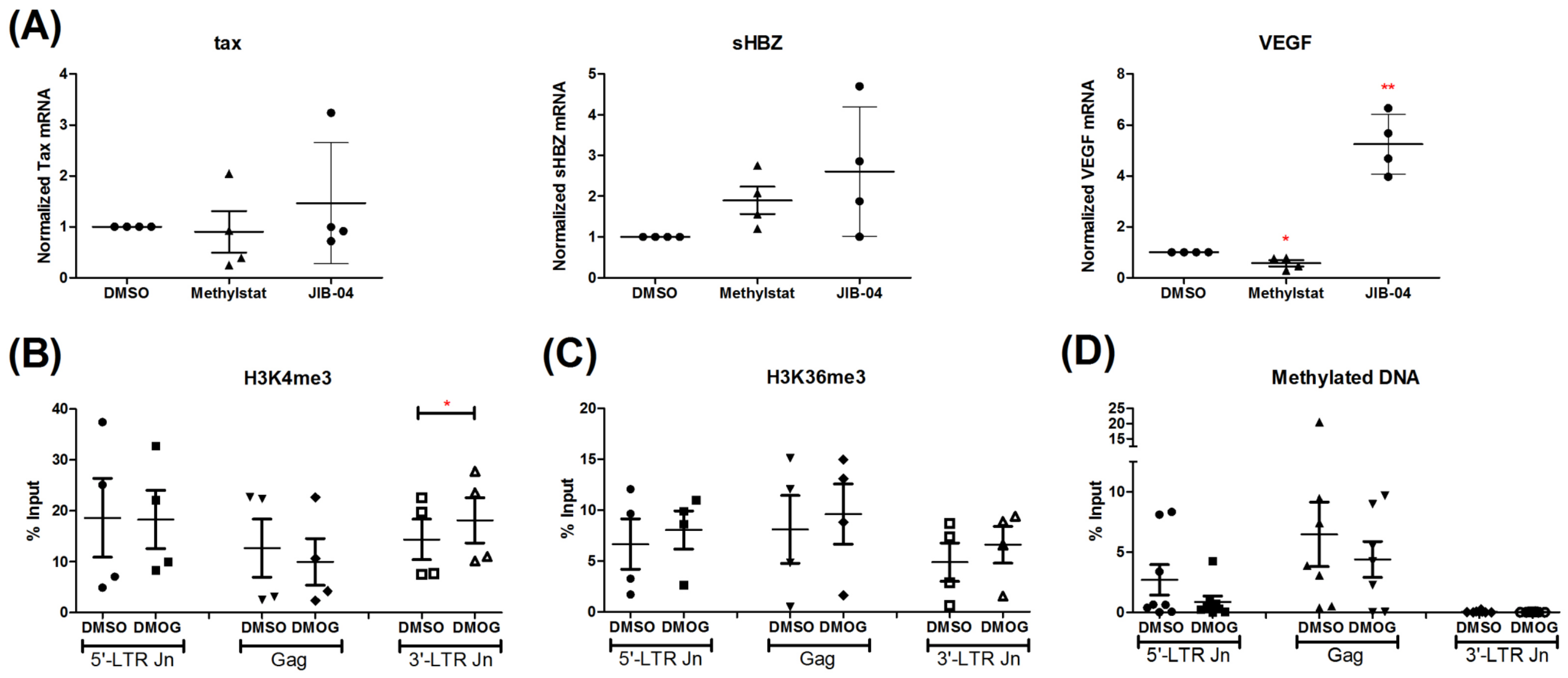


**Figure S3: 2-OG oxygenases do not control plus-strand HTLV-1 transcription. (A)** HTLV-1-infected primary PBMCs were treated overnight with either the broad spectrum JmjC KDM inhibitors Methylstat, JIB-04, or a DMSO control. RNA was extracted and subjected to RT-qPCR with primers specific for *tax* mRNA (plus-strand), *sHBZ* (minus-strand) or *VEGF* mRNA (positive control). Error bars represent the standard error of the mean (n=4). Statistical significance was calculated using the two-tailed Student's T-test where * represents $p<0.05$ and **

represents $p<0.005$. **(B, C) Epigenetic (histone methylation status) changes at the HTLV-1 provirus following DMOG treatment.** HTLV-1-infected PBMCs were cultured overnight with either DMOG or DMSO (control) and subsequently fixed and subjected to ChIP-qPCR with antibodies directed against H3K4me3, H3K36me3 and IgG and primers specific for the 5'-LTR Jn, *Gag* and 3'-LTR Jn of the HTLV-1 LTR. Enrichment is expressed as % input DNA. Error bars represent the standard error of 4 independent ChIP experiments. Statistical significance was calculated using the two-tailed Student's T-test where * represents $p<0.05$. **(D) Effect of DMOG treatment on DNA cytosine methylation within the HTLV-1 provirus.** HTLV-1-infected PBMCs were cultured with either DMOG or DMSO (control) overnight. Genomic DNA was isolated, sonicated and subsequently subjected to Methylated DNA Immunoprecipitation using the Methyl Collector Ultra kit (Active Motif) and primers specific for the 5'-LTR Jn, *Gag* and 3'-LTR Jn of the HTLV-1 LTR. Enrichment is expressed as % input DNA. Error bars represent the standard error of the mean for the 5'-LTR & 3'-LTR regions (n=8), and for the Gag region subjected to MeDIP (n=7).

**Figure S4. Related to Figures 5 and 6**

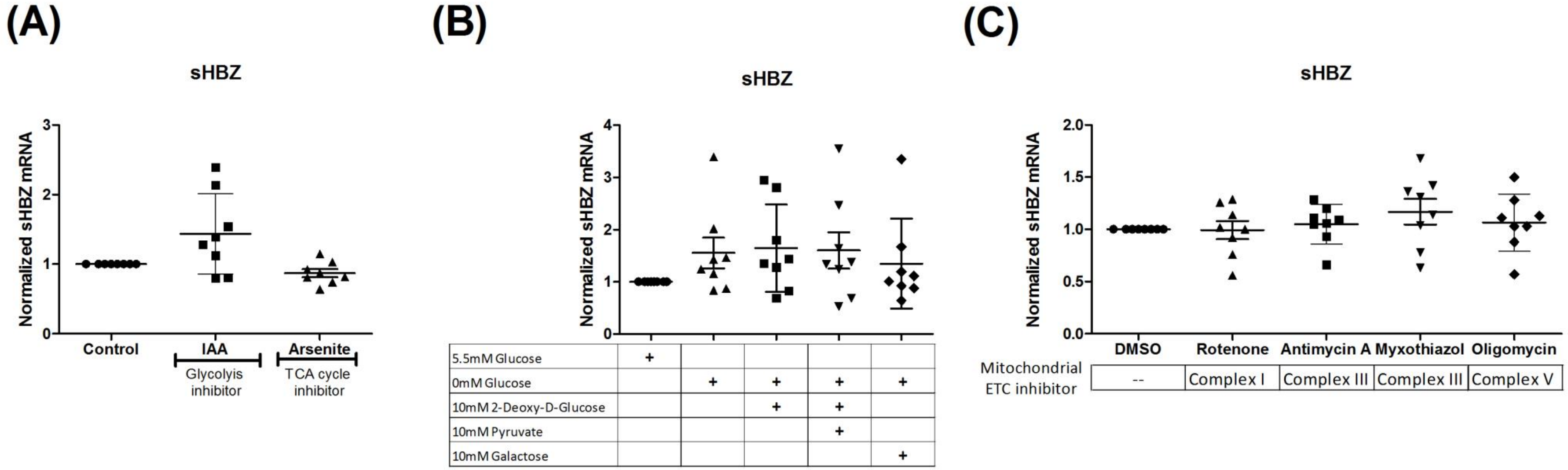


**Figure S4: Glucose metabolism and the mitochondrial electron transport chain do not govern minus-strand HTLV-1 transcription.** HTLV-1-infected primary PBMCs were treated overnight with control (water), iodoacetic acid (IAA) or sodium arsenite **(panel A)** or cultured under indicated concentrations of glucose-containing RPMI in the presence or absence of the glycolysis inhibitor 2-deoxy-D-glucose (2-DG) & TCA cycle inducers sodium pyruvate and galactose **(panel B)** or cultured with the indicated mitochondrial ETC inhibitors **(panel C)**. RNA was extracted and subjected to RT-qPCR with primers specific for *sHBZ* mRNA (minus-strand). Error bars represent standard error of the mean (N=8). Statistical significance was calculated using the two-tailed Student's T-test where * represents $p<0.05$.

**Table S1. Chemical compounds list. Related to all figures (1-6)**

| Sr. No. | Name | Supplier | Solvent | Final conc. | Mechanism of action | Ref |
|---|---|---|---|---|---|---|
| 1 | DMOG | Cayman Chem. | DMSO | 0.5mM | Broad spectrum 2-OG oxygenase inhibitor | (Jaakkola et al., 2001) |
| 2 | IOX2 | Cayman Chem. | DMSO | 50µM | Selective PHD inhibitor | (Chowdhury et al., 2013) |
| 3 | Iodoacetic acid (IAA) | Sigma | Water | 20µM | Glycolysis inhibitor, ROS inducer | (Sabri and Ochs, 1971) |
| 4 | Sodium Arsenite | Courtesy: CJS | Water | 1µM | TCA cycle inhibitor, ROS inducer | (Bergquist et al., 2009) |
| 5 | 2-Deoxy-D-Glucose | Sigma | Water | 10mM | Glycolysis inhibitor | (Zhong et al., 2009) |
| 6 | Sodium Pyruvate | Life tech. | Solution | 10mM | TCA cycle inducer | (Diers et al., 2012) |
| 7 | Galactose | Sigma | Water | 10mM | Oxphos inducer | (Aguer et al., 2011) |
| 8 | 3-Nitropropionic acid | Courtesy: CJS | DMSO | 1mM | ETC Complex 2 inhibitor | (Rohlena et al., 2013) |
| 9 | Rotenone | Sigma | Chloroform | 10µM | ETC Complex 1 inhibitor | (Rohlena et al., 2013) |
| 10 | Antimycin A | Sigma | DMSO | 10µM | ETC Complex 3 inhibitor | (Rohlena et al., 2013) |
| 11 | Myxothiazol | Sigma | DMSO | 10µM | ETC Complex 3 inhibitor | (Rohlena et al., 2013) |
| 12 | Oligomycin | Sigma | DMSO | 10uM | ATP synthase (ETC Complex V) inhibitor | (Rohlena et al., 2013) |
| 13 | Methylstat | Sigma | DMSO | 10µM | JmJc Histone Demethylase inhibitor | (Luo et al., 2011) |
| 14 | JIB-04 | Sigma | DMSO | 2µM | JmJc Histone Demethylase inhibitor | (Wang et al., 2013) |

**Table S2. List of primers and probes used for qRT-PCR, ChIP-qPCR and MeDIP. Related to all figures (1-6)**

| Sr. No | Gene | Orientation | Sequence | |
|---|---|---|---|---|
| **qRT-PCR** | | | | |
| 1 | Tax | F | 5′-CCGGCGCTGCTCTCATCCCGGT-3′ | (Satou et al., 2016) |
| | | R | 5′-GGCCGAACATAGTCCCCCAGAG-3′ | |
| 2 | sHBZ | F | 5′-GGACGCAGTTCAGGAGGCAC-3′ | |
| | | R | 5′-CCTCCAAGGATAATAGCCCG-3′ | |
| 3 | 18S rRNA | F | 5′-GTAACCCGTTGAACCCCATT-3′ | |
| | | R | 5′-CCATCCAATCGGTAGTAGCG-3′ | |
| | | | | |
| 4 | VEGF | F | 5'-GACTCCGGCGGAAGCAT-3' | (van Kuijk et al., 2016) |
| | | R | 5'-TCCGGGCTCGGTGATTTA-3' | |
| | | | | |
| | | | | |
| 5 | HMOX1 | F | 5′-CTCAAACCTCCAAAAGCC-3′ | (Reichard et al., 2007) |
| | | R | 5′-TCAAAAACCACCCCAACCC-3′ | |
| | | | | |
| | | | | |
| | | | | |
| 6 | LDHA | F | 5′-TTGGTCCAGCGTAACGTGAAC-3′ | (Allison et al., 2014) |
| | | R | 5′-CCAGGATGTGTAGCCTTTGAG-3′ | |
| | | | | |
| | | | | |
| **ChIP-qPCR and MeDIP** | | | | |
| | | | | (Satou et al., 2016) |
| 1 | 5'-LTR Junction | F | 5′-GACAGCCCATCCTATAGCACTC-3′ | |
| | | R | 5′-CTAGCGCTACGGGAAAAGATT-3′ | |
| 2 | Gag | F | 5′-CAGAGGAAGATGCCCTCCTATT-3′ | |

| | | | | |
|---|---|---|---|---|
| | | R | 5′-GTCAACCTGGGCTTTAATTACG-3′ | (Satou et al., 2016) |
| 3 | Pol | F | 5'-CAGCCCATTCGGCAAG-3' | |
| | | R | 5'-TGAGAGTAGTAGTAGGTCCTCATGG-3' | |
| 4 | Env | F | 5'-GCTAGTTCTGCCCAGTGGAT-3' | |
| | | R | 5'-TTGGTGGTCTTTTTCTTTGG-3' | |
| 5 | vCTCF | F | 5'-CCGATCACGATGCGTTTC-3' | |
| | | R | 5'-ACGGTTTGCTATCCTTAGAAGA-3' | |
| 6 | Tax | F | 5'-CTCCTTCCGTTCCACTCAAC-3' | |
| | | R | 5'-GTGGTAGGCCTTGGTTTGAA-3' | |
| 7 | 3'-LTR Junction | F | 5′-AATACACCAACATCCCCATTTC-3′ | |
| | | R | 5′-GTTTTTCACTGGGAGGCTCTAA-3′ | |
| | | | | |
| **qPCR Probes (ChIP-qPCR and MeDIP)** | | | | |
| | | | | |
| 1 | 5'-LTR Junction | | FAM -TCGTCCGGGATACGAGCGCC- TAM | |
| 2 | Gag | | FAM -TACCGTTAGATCCCGCCCGTCG- TAM | |
| 3 | Pol | | FAM- CATGGATGACATTCTCCTAGCAAGCCC - TAM | |
| 4 | Env | | FAM-CTGCATGCCCAAGACCCGTCG-TAM | |
| 5 | vCTCF | | FAM-TGGAGGGCCCCGTCGCA-TAM | |
| 6 | Tax | | FAM-CGCCTATGATTTCCGGGCCCTG-TAM | |
| 7 | 3'-LTR Junction | | FAM -AGAGGCAGATGACAATGACCATGAGCC- TAM | |
| | | | | |
| | | | | |
| | | | | |
